# Participation profile and environmental context of brazilian children with cerebral palsy: associations with functioning and environmental factors

**DOI:** 10.1590/1984-0462/2025/43/2025014

**Published:** 2025-12-15

**Authors:** Viviann Alves de Pontes, Jaíza Marques Medeiros e Silva, Nadine Oliveira Cabral, Kennea Martins Almeida Ayupe, Rosane Luzia de Souza Morais, Paula Silva de Carvalho Chagas, Hércules Ribeiro Leite, Rafaela Silva Moreira, Ana Carolina de Campos, Aline Martins de Toledo, Ana Cristina Resende Camargos, Hemílio Fernandes Campos Coêlho, Egmar Longo

**Affiliations:** a Universidade Federal da Paraíba, João Pessoa, PB, Brasil.; b Universidade de Brasília, Brasília, DF, Brasil.; c Universidade Federal do Espírito Santo, Vitória, ES, Brasil.; d Universidade Federal dos Vales do Jequitinhonha e Mucuri, Diamantina, MG, Brasil.; e Universidade Federal de Juiz de Fora, Juiz de Fora, MG, Brasil.; f Universidade Federal de Minas Gerais, Belo Horizonte, MG, Brasil.; g Universidade Federal de Santa Catarina, Araranguá, SC, Brasil.; h Universidade Federal de São Carlos, São Carlos, SP, Brasil.

**Keywords:** Child health, Social participation, Social environment, Cerebral palsy, Saúde da criança, Participação social, Ambiente social, Paralisia cerebral

## Abstract

**Objective:**

The objective of this study was to explore community participation and environmental profiles of young Brazilian children with CP and analyze the association between participation domains, environmental factors, and the child’s GMFCS level.

**Methods:**

Cross-sectional study derived from the “PartiCipa Brasil” project, involving 109 Brazilian children with CP, with a mean age of 44.8 months (SD=12.0). The sociodemographic questionnaire characterized the sample, the Gross Motor Function Classification System (GMFCS) — Family Report classified the functionality, and the Young Children’s Participation and Environment Measure (YC-PEM) assessed participation and environmental factors in the community. The data were analyzed using descriptive statistics and Spearman correlation to investigate relationships between the domains of participation, environmental factors, and the GMFCS, adopting p<0.05 as significance.

**Results:**

Male predominance, spastic CP, and GMFCS level V, with children from low-income families. Children participated in the community “few times in the last month” and were involved at a “between 3 and 5” level, with an emphasis on activities related to routine appointments. There was a higher level of support compared to the barriers faced, and 49.8% of caregivers expressed a desire to change their participation. The child’s environmental factors and their GMFCS level correlated with their participation.

**Conclusions:**

This is the first study using data from PartiCipa Brasil that describes how young children with CP from different regions of Brazil participate in the community. Understanding participation and environmental factors can support the creation of interventions to improve opportunities for this public to participate in society.

## RESUMO

**Objetivo:**

Descrever a participação na comunidade e o perfil ambiental de crianças pequenas brasileiras com paralisia cerebral (PC) e analisar a correlação entre os domínios de participação, fatores ambientais e o nível da criança pelo Gross Motor Function Classification System (GMFCS).

**Métodos:**

Estudo transversal derivado do projeto “PartiCipa Brasil”, que envolveu 109 crianças brasileiras com PC, com idade média de 44,8 meses (desvio padrão — DP=12,0). O questionário sociodemográfico caracterizou a amostra, o GMFCS-Family Report classificou a funcionalidade, e o Young Children’s Participation and Environment Measure (YC-PEM) avaliou a participação e os fatores ambientais na comunidade. Os dados foram analisados por estatística descritiva e correlação de Spearman para investigar relações entre os domínios de participação, fatores ambientais e o GMFCS, adotando p<0,05 como significância.

**Resultados:**

Predomínio do sexo masculino, PC espástica e nível V do GMFCS, com crianças oriundas de famílias de baixa renda. As crianças participaram da comunidade “algumas vezes no último mês” e se envolveram no nível “entre 3 e 5,” com ênfase em atividades relacionadas a compromissos de rotina. Observou-se maior nível de “suportes” em comparação às “barreiras”, e 49,8% dos cuidadores expressaram “desejo de mudança” na participação. Os fatores ambientais e o nível de GMFCS da criança correlacionaram-se com sua participação.

**Conclusões:**

Trata-se do primeiro estudo com dados do PartiCipa Brasil que descreve como crianças pequenas com PC de diferentes regiões do Brasil participam na comunidade. Compreender a participação e os fatores ambientais pode apoiar a criação de intervenções para melhorar as oportunidades de participação deste público na sociedade.

## INTRODUCTION

The initial years of life are crucial for child development and are shaped by experiences in supportive environments that influence the child’s participation profile in family, school, and community contexts.^1^ The International Classification of Functioning, Disability, and Health (ICF) defines participation as involvement in everyday life situations.^2^ To facilitate its application, this concept has been expanded through the family of participation-related constructs (fPRC), which proposes understanding participation based on two components: attendance and involvement.^3^ The evolution of the participation concept has significantly contributed to the advancement of clinical practice and research. The ICF also defines the environment as the set of physical, social, and attitudinal factors that may act as barriers or facilitators to the child’s participation in different activities.^2^

Participation is an important health outcome, especially for young children with disabilities, who often face more barriers to participation compared to their non-disabled peers. This usually happens in community contexts for those under 6 years of age, with these constraints being particularly pronounced in community settings.^4,5,6,7,8^ Therefore, their caregivers often express dissatisfaction regarding these limitations and participation restrictions.^9,10^

Cerebral palsy (CP) is the most common physical disability in pediatric populations.^11^ In Brazil, children with CP exhibit significant impairments in gross motor function, evaluated using the Gross Motor Function Classification System (GMFCS).^12^ Research indicates that young children, from infancy up to 6 years old,^13^ with CP face significant restrictions in community participation.^5,8^ This is often associated with child, family, and environmental factors.^8,14^ Current research on the participation of young children with disabilities, including CP, in community settings has been conducted primarily in high-income countries.^5,6,8,14,15^ According to these studies, the main barriers to participation for young children with disabilities are age, limitations in motor skills, environmental usefulness, and family functioning.

Cultural variability often has a crucial role in influencing participation levels,^16^ underscoring the importance of investigating this outcome in middle- and low-income countries. In Brazil, a recent study with young children with myelomeningocele, using the Young Children’s Participation and Environment Measure (YC-PEM), identified that age was the main factor associated with the number of activities carried out in the community, while mobility influenced involvement and the desire for changes in community participation.^17^ Nonetheless, the literature

on participation in CP remains limited, often constrained by small sample sizes,^18^ a focus predominantly on children over 5 years old,^19^ as well as adolescents and young adults,^20^ or a failure to comprehensively examine all participation and environment domains of the fRPC.^21^ These limitations highlight the importance of expanding studies on the participation of young children with CP in the Brazilian context, addressing issues related to barriers, facilitators, and personal aspects. Therefore, this study aims to explore community participation and environmental profiles of young Brazilian children with CP and analyze the association between participation domains, environmental factors, and the child’s GMFCS level.

## METHOD

This is a cross-sectional, descriptive, and exploratory, quantitative study derived from the multicenter longitudinal research project entitled “Study protocol: functioning curves and trajectories for children and adolescents with cerebral palsy in Brazil–PartiCipa Brazil,”^22^ which involved children and adolescents with CP. The Research Ethics Committee of the Federal University of Juiz de Fora approved the study, which is registered under protocol number 5.832.326 (CAAE 28540620.6.1001.5133). Additional details regarding the study protocol can be accessed as needed.^22^

Participants were recruited from clinical settings at federal universities in Brazil, including university hospitals and rehabilitation centers, and through social media. The study sample was determined through a sample calculation based on the study of Di Marino et al.,^14^ considering a margin of error set at 0.13 and a confidence level of 95%, obtaining a minimum sample size of 102 children. Children aged 2–5 years and 11 months diagnosed with CP of both sexes, regardless of clinical type, whose caregivers signed the Informed Consent Form, were included. Children with motor disabilities resulting from other health conditions, such as myelomeningocele or genetic syndromes, or with incomplete data were excluded.

The children’s caregivers completed three assessment tools: a sociodemographic questionnaire; the GMFCS — Family Report;^23,24^ and the YC-PEM.^25^ These assessments were administered online using Google Forms.

Participants had two options for completion:

1. They could respond via a link shared through WhatsApp, with support from the research team available for any questions, or
2. They could engage in a videoconference interview with a member of the research team.

All examiners were physical therapists and received robust training in the administration of these instruments from a senior researcher associated with the CanChild group, who contributed to the development of YC-PEM. In addition, they followed a structured script to ensure the standardized delivery of questions in both modalities.

The sociodemographic questionnaire, developed by the PartiCipa Brasil group based on the International Classification of Functioning, Disability, and Health (ICF),^2^ was designed to gather information on the child’s personal and environmental factors. The GMFCS categorizes mobility into five levels, from I to V, where children at level I are independent in walking and mobility, and those at level V are completely dependent and require assistance.^23^ This study used the family report version of the GMFCS for children aged 2–4 and 4–6 years, both translated into Brazilian Portuguese.^26^ The version used showed inter-observer reliability and agreement ranging from moderate to strong (Kappa≥0.7).^27^

The YC-PEM assesses caregivers’ perceptions of the participation and environmental factors of children aged 0–5 years and 11 months, with or without disabilities, over the preceding four months.

This instrument comprises two scales:

1. Participation and
2. Environment, divided into three sections: home, day-care/preschool, and community, resulting in a total of 73 items.

The details of the YC-PEM domains and scores are in Table 1. We used the Brazilian translated and adapted version,^28^ which showed acceptable initial measurement properties.^29^

**Table 1:** Description of items in the participation and environment measure – Young Children (YC-PEM)^4^.

Scale/number of items	Domains	Scale score	Score
Participation Home: 13 items Daycare/preschool: 3 items Community: 11 items	Frequency	8 point scale (0–7) (0=Never; 1=Once in the last four months; 2=Few times in the last four months; 3=Once in the last month; 4=Few times in the last month; 5=Few times in the last month; 6=Few times each week; 7=Once or more each day)	Average frequency
	Involvement	5 point scale (1–5) (1=Not very involved; 2=Between 1 and 3; 3=Somewhat involved; 4=Between 3 and 5; 5=Very involved)	Average involvement
	Desire of Chang	2 point scale (0–1) (0=No; 1=Yes, you want to change)	Percentage of activities in which change is desired (0–100%)
Environment Home: 13 items Daycare/preschool: 16 items Community: 17 items	Supports	“Usually helps” or “Usually, yes”	Percentage of items classified as Supports to participation (0–100%)
	Barriers	“Usually makes harder” or “Usually, no”	Percentage of items classified as Barriers to participation (0–100%)
	Environmental Helpfulness	3 point scale: 1=Usually makes harder; 2=Sometimes helps; sometimes makes harder; 3=Usually helps; and No impact	Percentage of environmental helpfulness (0–100%)
	Environmental Resources	3 point scale: 1=Usually, no; 2=Sometimes yes, sometimes no; 3=Usually, yes; and Not needed	Percentage of environmental resources (0–100%)
	Overall Environmental Support	All environmental scores	Percentage of overall environmental support (0–100%)

In this study, we examined participation and environment within the community section of the translated Brazilian version of the YC-PEM,^28^ because this section is less explored in the literature and focuses on social and leisure activities, key determinants of the health of young children with CP.^5,14,16^ The Desire for Change was categorized into three distinct groups: Frequency, which included responses such as “Yes, do more often” and “Yes, do less often”; Involvement, signifying answers like “Yes, be more interactive” and “Yes, be more helpful”; and Variety of Activities, represented by the response option “Yes, participate in a broader variety of activities.”

The scores for all the domains of the Brazilian version of the YC-PEM were computed using a customized calculator (Excel) created following the guidelines and options of the original version of the YC-PEM.^30^

The data were analyzed through descriptive statistics, including median, absolute values, percentages, and interquartile ranges. A descriptive analysis was made of participation (frequency, involvement, desire for change, and types of desire for change) and also of each activity per item assessed (e.g., shopping and errands, dining out). For the descriptive analysis of the Environment section by item assessed by YC-PEM, the percentage of 40% will be considered a significant cut-off point if all caregivers opt for this option, according to the study by Krieger et al.^31^ Spearman’s correlation test was used to investigate the association between the participation domains (frequency, involvement, and desire for change) and the participant’s GMFCS level, as well as with the environment domains (supports, barriers, environmental resources, environmental helpfulness, and overall environmental support). The interpretation of Spearman’s test was as follows: correlations of 0.00–0.30 were considered insignificant; 0.30–0.50, a weak correlation; 0.50–0.70, a moderate correlation; 0.70–0.90, a high correlation; and 0.90–1.00, a very high correlation.^32^ A significance level of <0.05 was established for all analyses. The statistical analyses were conducted using Jamovi software (v.2.3.28).

## RESULTS

**Figure 1.**
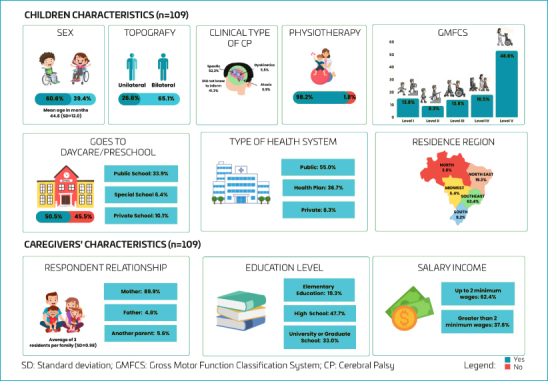
Descriptive characteristics of the participants in this study.

Of 541 children with CP monitored by the PartiCipa Brasil project from July 2021 to July 2024, 128 families completed the YC-PEM questionnaire. Of these, 19 were excluded due to incomplete GMFCS data. As a result, 109 Brazilian children participated in this study. Figure 1 displays the characteristics of the study participants. It was found that the children had a mean age of 44.8 months (SD=12.0), were mostly males, had spastic CP, and were classified as GMFCS level V.

The participants reported a median YC-PEM frequency score of 4.0, indicating participation “a few times in the last month,” and an involvement score of 4.0, which suggests a level corresponding to “between 3 and 5.” They participated in 7 of the 11 possible activities, and 49.8% of caregivers expressed a desire for change. When considering the environment, more participants perceived supports (52.8%) than barriers (19.3%) (Table 2).

**Table 2:** Descriptive results of Community Participation and Environment according to the Participation and Environment Measure – Young Children (YC-PEM) and Spearman Correlations with GMFCS and Environmental Factors

YC-PEM subscale			Children (n=109)				
			Median		(Q1–Q3)	
Participation				
Frequency (0–7)			4.0		3.0–5.0	
Involvement (1–5)			4.0		3.0–5.0	
Desire for change (%)			45.0		18.0–82.0	
Number of activities (0–11)			7.0		6.0–8.0	
Environment						
Supports (%)			53.0		35.0–71.0	
Barriers (%)			12.0		6.0–29.0	
Environmental helpfulness (%)			77.0		70.0–90.0	
Environmental resources (%)			89.0		76.0–94.0	
Overall environmental support (%)			82.0		73.0–88.0	
**Variables**	**Domains of participation in the YC-PEM community section**					
	**Frequency**		**Involvement**		**Desire for change**	
	**r^2^**	**p-value**	**r^2^**	**p-value**	**r^2^**	**p-value**
GMFCS	-0.240	0.011*	0.031	0.748	0.184	0.054
Supports	0.099	0.304	0.230	0.016*	-0.274	0.003*
Barriers	-0.024	0.799	-0.158	0.099	0.340	<0.001*
Environmental Helpfulness	0.106	0.272	0.210	0.028*	-0.359	<0.001*
Environmental resources	-0.017	0.852	0.091	0.343	-0.159	0.097
Overall environmental support	0.092	0.336	0.238	0.012*	-0.344	<0.001*

GMFCS: Gross Motor Function Classification System.

*p<0.05.

In the correlation analyses (Table 2), there is a weak, negative, and statistically significant correlation between GMFCS and the frequency of participation. At the level of involvement of participation, weak, positive, and statistically significant correlations were noted with supports, environmental helpfulness, and overall environmental support. Regarding the desire for change, a weak, positive, and statistically significant correlation was observed with barriers, while negative correlations were identified with supports, environmental helpfulness, and overall environmental support.

Concerning activity participation by item, as shown in Table 3, it was observed that there was a higher frequency of participation and involvement in the activity of “routine appointments” (i.e., “medical appointments, therapies, and trips to the dentist”) and a lower frequency and involvement in “classes and lessons” (i.e., “extracurricular classes such as music and languages”). As for the desire for change expressed by caregivers, the greatest dissatisfaction refers to children’s participation in “community attractions” (i.e., “trips to the bookstore, cinema, or zoo”). Conversely, the activity that generated the most satisfaction, with the least desire for change, was “shopping and errands” (i.e., “trips to the market, shopping mall, and bakery”).

**Table 3:** Frequency, Involvement, Desire for change, and types of desire for change in the Community based on the Participation and Environment Measure – Young Children (YC-PEM).

	Frequency		Involvement		DC		Types of desire for change – n (%)		
	Med	(Q1–Q3)	Med	(Q1–Q3)	%	n*	Frequency	Involvement	Variety
Activities (n=109)
Shopping and errands	5.0	4.0–6.0	4.0	3.0–5.0	37.6	46	19 (41.30)	17 (36.96)	10 (21.74)
Dining out	4.0	2.0–5.0	4.0	1.0–5.0	45.0	56	22 (39.29)	22 (39.29)	12 (21.43)
Routine appointments	6.0	5.0–6.0	5.0	3.0–5.0	42.2	55	18 (32.73)	23 (41.82)	14 (25.45)
Classes and lessons	0.0	0.0–0.0	0.0	0.0–0.0	46.8	54	21 (38.89)	7 (12.96)	26 (48.15)
Organized physical activities	0.0	0.0–5.0	0.0	0.0–5.0	58.7	76	30 (39.47)	9 (11.84)	37 (48.68)
Community attractions	0.0	0.0–2.0	1.0	0.0–4.0	62.4	75	30 (40.00)	15 (20.00)	30 (40.00)
Religious or spiritual gatherings and activities	3.0	0.0–5.0	3.0	1.0–5.0	49.5	58	30 (51.72)	19 (32.76)	9 (15.52)
Social gatherings	3.0	2.0–4.0	3.0	3.0–5.0	48.6	63	22 (34.92)	27 (42.86)	14 (22.22)
Community events	0.0	0.0–2.0	1.0	0.0–5.0	42.2	54	23 (42.59)	13 (24.07)	18 (33.33)
Unstructured activities	4.0	2.0–5.00	5.0	3.0–5.0	56.9	71	33 (46.48)	18 (25.35)	20 (28.17)
Overnight visits or trips	1.0	0.0–2.0	3.0	0.0–5.0	56.0	69	38 (55.07)	11 (15.94)	20 (28.99)

n: number; Med: median; Q1–Q3: interquartile range; DC: desire for change.

*number of responses

Note: Due to the multiple response options, the percentages of the types of desire for change refer to the total responses received.

In Table 3, when analyzing the types of desired change, a higher percentage of individuals expressing a desire for change regarding frequency was found for “overnight visits or trips,” while involvement related to “social gatherings” (i.e., “birthday parties or group games”). Regarding the desire for change concerning the variety of activities, there was a higher preference for “organized physical activities” (i.e., “soccer, dancing, or swimming”).

**Figure 2.**
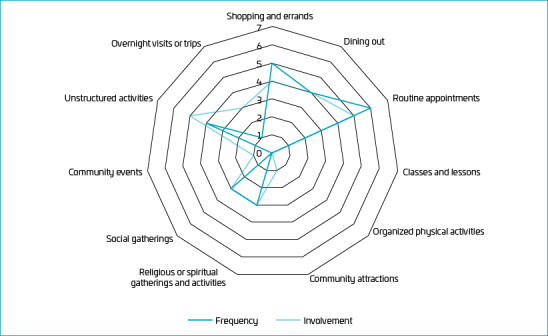
Radar chart of participation frequency and involvement in the community section.

The median scores for frequency and involvement in the community section are illustrated using a radar chart in the format of hendecagon, that is, an 11-sided polygon representing the 11 activities included in this section (Figure 2). The scores are displayed in descending order (ranging from 7 to 0), with each line denoting a specific score level. A score further away from the origin indicates a better performance. In this study, the activities that garnered the highest levels of participation were “shopping and errands” and “routine appointments.”

Regarding environmental aspects in the community, as seen in Table 4, caregivers identified relationships with peers, the “social demands of activities,” and “people’s attitudes toward the child” as supportive factors, “access to personal transport,” “equipment or supplies,” “time,” and “information” were recognized as resources. The primary barriers recognized were “physical layout,” “safety,” and “sensory quality.” Within the resources section, some caregivers indicated that “information,” “money,” and “programs and services” posed challenges.

**Table 4:** Environmental aspects in the YC-PEM community section.

(total n=109 children)	Supports		Barriers	
	n	%	n	%
Factors:
Do the following aspects of the community environment help or hinder a child’s participation in community activities?	**“Usually helps”**		**“Usually makes harder”**	
1. Physical layout or amount of space outside and inside the buildings	26	23.9	34	31.2
2. Sensory qualities of community settings	14	12.8	30	27.5
3. The physical demands of typical activities	26	23.9	24	22.0
4. The cognitive demands of typical activities	29	26.6	21	19.3
5. The social demands of typical activities	49	45.0*	11	10.1
6. The attitudes and actions of other members of the community towards your child	46	42.2*	7	6.4
7. Your child’s relationships with peers	69	63.3*	7	6.4
8. Outside weather conditions	13	11.9	24	22.0
9. The safety of the community	27	24.8	32	29.4
10. Policies	29	26.6	13	11.9
Resources:
Are the following available or appropriate to help your child participate in the community?	**“Usually, yes”**		**“Usually, no”**	
11. Access to personal transportation	68	62.4*	16	14.7
12. Access to public transportation	41	37.6	22	20.2
13. Programs and services	38	34.9	27	24.8
14. Equipment or supplies	67	61.5*	15	13.8
15. Information	47	43.1*	35	32.1
16. Time	67	61.5*	12	11.0
17. Money	33	30.3	26	23.9

*Significant if more than 40% of all caregivers chose this option. The cutoff point was adopted according to the study by Krieger et al., 2024^10^.

Shopping and errands

## DISCUSSION

To our knowledge, this study is the first to describe community participation and environmental factors in young Brazilian children with CP. The majority of the participants are low-income, have greater impairment of gross motor function, have access to public health, mainly physiotherapy, half attend daycare centers, and live mostly in the southeast of Brazil. These personal and environmental factors likely influenced our results, as physical, social, and economic barriers significantly impact engagement in community activities.

The severity of motor impairment is a key determinant of participation, and in developing countries like Brazil, limited access to early CP diagnosis and intervention contributes to a higher proportion of individuals with severe impairment.^33^ Additionally, socioeconomic disadvantages further constrain opportunities for participation, limiting access to structured activities, transportation, and community-based resources.^34,35^ The role of the environment is particularly critical for preschool children with higher levels of motor impairment, as contextual adaptations can either facilitate or hinder engagement in meaningful activities.^36^

We found that participants were generally with high involvement, close to the maximum of the scale, and engaged “few times in the last month.” Half of the caregivers desired change, with more perceiving supports than barriers in their environment. Our findings suggest that the YC-PEM environmental domains, alongside with the level of gross motor function are correlated with participation domains. These results prompt further inquiry into aspects beyond physical disabilities, including individual, family, and environmental characteristics, which serve as social determinants of health.

The children in this study demonstrated a frequency of community participation described as “a few times in the last month.” This frequency which was notably higher than that reported in previous studies involving children with disabilities and a similar age group carried out in China,^7^ Canada,^14^ and the United States,^4^ where participation was typically described as “once in the last month.” Additionally, this frequency exceeds that reported in high-income countries like Singapore and Lithuania, where participation was “a few times in the last four months”.^6,8^ In this study, the children showed a level of Involvement “between 3 and 5,” indicating a score close to the maximum on the scale and slightly higher than that reported in previous research from high-income countries,^7,14^ suggesting that Brazilian young children with CP can effectively engage in community activities.

The participation results in this study may be influenced by the sociocultural context. Notably, the activity with the highest participation was “routine appointments” which contrasts with findings reported in middle- and high-income coun- tries,^8,14^ where the most frequent activities were “shopping and errands” and “unstructured activities.” This level of participation was anticipated given that a majority of the sample has severe motor impairments and nearly all of them undergo physiotherapy, which often requires a considerable investment of time.^8^

Alternatively, in the current sample, the activities with the lowest participation were “classes and lessons.” This finding contrasts with the study by Lim et al.^6^ conducted in a high-income country, where the least frequently reported activities were “overnight visits or trips.” The low participation rates of Brazilian children with CP in “classes and lessons” can be largely attributed to economic constraints faced by their families, limiting investment in developmental opportunities. This situation may also be worsened by inadequate accessibility and lack of inclusion in extracurricular activities.^9^

We identified that participation in certain activities, such as “community attractions” (including bookstores, cinemas, and zoos), “community events” (including festivals, performances, or games), “overnight visits or trips,” as well as “unstructured activities” (such as soccer, swimming, and dancing), tends to be low. This diminished participation may be largely attributed to the financial constraints faced by many individuals in the sample, which aligns with the lower income levels identified. This factor has been recognized as a predictor of participation in prior research.^34,35^

The literature shows heterogeneous findings in the reported desire for change.^4,6,7,14^ This variation may stem from cultural differences, contextual challenges, and varying parental preferences across the studies. In the current study, approximately half of the caregivers expressed a desire for change, suggesting dissatisfaction with the activities their children participate. Di Marino et al.^14^ observed a low desire for change among caregivers, which the authors attribute to differing expectations from parents regarding the community participation of their children with disabilities, an expectation that may evolve as children acquire more skills.

No studies in the existing literature have focused on young children with CP and their desire for change in activities, highlighting an area that warrants further exploration. In this study, the activity that showed the greatest desire for change was visiting “community attractions,” which also exhibited some of the lowest frequencies and levels of involvement. Caregivers expressed a desire for increased frequency in travel-related activities, more active involvement in “social gatherings,” and a broader variety of “organized physical activities.” These findings indicate that, despite limited opportunities, there is a strong yearning for enriching experiences that foster children’s socialization and overall development.

Regarding the environmental factors experienced by the sample in the community section, “relationships with peers,” “attitudes,” and “social demands of activities” emerged as supports, underscoring the significance of social interactions in children’s lives. Previous research backs this assertion up, indicating that community participation fosters social interaction, which enhances emotional well-being, social skills, and the development of identity and autonomy.^16,37^ Furthermore, access to personal transportation, necessary equipment, and supplies, as well as the availability of time and information were identified as supports. These findings emphasize the importance of integrating components that can optimize outcomes in children’s participation.

Contrarily, the barriers identified by caregivers, including issues related to “physical layout” and “safety,” are concerning. Environments that are neither accessible nor secure can hinder children’s participation in activities, ultimately hampering their development.^1^ Additionally, sensory quality has emerged as a significant barrier; environments characterized by excessive noise, harsh lighting, or uncomfortable temperatures can lead to discomfort and diminish active participation.^38^ These factors reinforce the necessity for environmental adaptations to promote participation and development of these children.

The results reveal several weak yet significant correlations between the variables. These results could potentially be attributed to the small sample size, especially given the considerable variability of the characteristics of the participants. The negative correlation observed between GMFCS and the frequency of participation suggests that children with greater motor limitations demonstrate a reduced level of participation in activities, which is consistent with previous literature.^5,37^ However, the observed correlation was not strong, suggesting that determinants beyond motor function significantly influence participation frequency.^35^

The positive correlations between supports and environmental helpfulness and a child’s involvement suggest that an environment rich in supports promotes greater participation in activities. This finding is confirmed by the research conducted by Anaby et al.,^39^ indicating that community participation is influenced by both supports and barriers. The desire for change showed positive correlation with barriers and negative correlations with environmental supports, environmental helpfulness, and overall environmental support. This underscores the necessity to modify or enhance the community environment to align with the changes desired by caregivers and to improve the participation of children with CP.

This study has certain limitations, including the challenge of comparing participation profiles with findings from previous research due to the varied diagnoses within the samples and the collective analysis of results alongside non-disabled groups. The method of instrument administration may present limitations, whether through self-response links or videoconference, particularly considering potential differences in comprehension and response patterns among participants with varying education levels, as well as the cross-sectional nature of the study prevents the establishment of causal relationships. Moreover, we acknowledge that the findings of this study should be interpreted within the context of this specific population, as these demographic and clinical characteristics may limit the broader applicability of the results. Nevertheless, these limitations did not undermine the results, as the study’s objectives were successfully met.

In conclusion, the Brazilian children with CP included in this study participate more frequently in community activities associated with “routine commitments” while showing less involvement in “classes and lessons.” A greater level of support was noted when compared to the barriers faced. Caregivers expressed varying degrees of desire for change, with notable dissatisfaction concerning “community attractions.” The child’s environmental factors and their GMFCS level correlated with their participation. It is suggested that future studies involve larger sample sizes to explore the factors influencing participation and to further investigate the desire for change domain, to develop targeted interventions to enhance participation.

These findings provide an empirical basis for developing and implementing evidence-based interventions that prioritize participation and environmental modifications tailored to different levels of functioning. Furthermore, prioritizing community engagement of children with CP and their families may increase the ecological validity of rehabilitation strategies by promoting personalized approaches that optimize functional outcomes and long-term participation in meaningful activities.

## Data Availability

The database that originated the article is available with the corresponding author.
